# Cognitive, Emotional, and Daily Functioning Domains Involved in Decision-Making among Patients with Mild Cognitive Impairment: A Systematic Review

**DOI:** 10.3390/brainsci14030278

**Published:** 2024-03-14

**Authors:** Federica Alfeo, Tiziana Lanciano, Chiara Abbatantuono, Giorgia Gintili, Maria Fara De Caro, Antonietta Curci, Paolo Taurisano

**Affiliations:** 1 Department of Education, Psychology, Communication, University of Bari “Aldo Moro”, 70122 Bari, Italy; federica.alfeo@uniba.it (F.A.); antonietta.curci@uniba.it (A.C.); 2 Department of Translational Biomedicine and Neuroscience (DiBraiN), University of Bari “Aldo Moro”, 70124 Bari, Italy; chiara.abbatantuono@uniba.it (C.A.); giorgiagin97@gmail.com (G.G.); paolo.taurisano@uniba.it (P.T.)

**Keywords:** MCI, neurocognitive disorders, decision-making, memory and learning, emotion, executive functions, systematic review

## Abstract

Mild cognitive impairment (MCI) is a transitional or prodromal stage of dementia in which autonomies are largely preserved (autonomies are not particularly affected). However, this condition may entail a depletion of decision-making (DM) abilities likely due to a gradual deterioration of the prefrontal cortex and subcortical brain areas underlying cognitive–emotional processing. Given the clinical implications of a decline in self-determination observed in some MCI sufferers, the present systematic review was aimed at investigating the literature addressing DM processes in patients with MCI, consistent with PRISMA guidelines. The six online databases inquired yielded 1689 research articles that were screened and then assessed based on eligibility and quality criteria. As a result, 41 studies were included and classified following the PICOS framework. Overall, patients with MCI who underwent neuropsychological assessment were found to be slightly or moderately impaired in DM abilities related to financial management, medical adherence, specific cognitive performances, risky conditions, and especially uncertain life circumstances. Comparative cross-sectional studies indicated not only mid-stage cognitive functioning in MCI but also borderline or deficit DM patterns evaluated through different tasks and procedures. Further research addressing MCI profiles suggested an association between explicit memory, executive functions, and DM performance. These findings highlight the diversity of MCI manifestations, in addition to the critical importance of DM features and correlates in patients’ daily functioning. Due to a lack of consensus on both MCI and DM, this review paper sought to shed light on assessment and intervention strategies accounting for the interplay between emotion, motivation, and learning to foster DM in cognitively impaired individuals.

## 1. Introduction

### 1.1. General Background

Mild cognitive impairment (MCI) is a transitional stage between healthy and pathological neurocognitive aging characterized by objective evidence of decline in one or more cognitive domains, which do not interfere with one’s functional independence [1,2]. Due to the clinical heterogeneity of MCI, patients diagnosed with this condition can be classified into four subtypes according to the presence or absence of episodic memory deficits (i.e., amnestic vs. non-amnestic MCI), and the extent of impaired cognitive domains (i.e., single- vs. multiple-domain MCI) [3,4]. Although estimates of MCI prevalence vary widely by patients’ age, MCI sub-type, and the severity of cognitive decline [5], it is currently reckoned that 10 to 15% of the global population aged over 65 years is affected by such cognitive dysfunction, thus requiring clinical monitoring [6].

It should be noted that, over time, MCI sufferers may revert to normal cognition, remain stable, or convert to an obvious neurocognitive disorder [7,8]. Consistently, a longitudinal, history-based approach may be recommended to accurately predict the progression trajectories of MCI. For instance, individuals with amnestic MCI are likely to develop Alzheimer’s dementia when further risk factors (e.g., family history of Alzheimer’s disease [AD]; biomarkers of Aβ deposition and neural injury; comorbidities) are involved [6,9]. Among these individuals, additional cognitive abilities, notably the executive functions (EFs) of inhibitory control were found to be modestly impaired [10]. Moreover, poorer performances in executive tasks seem to be predictive of a decline in instrumental activities of daily living, suggesting higher risks for MCI-to-dementia conversion [11].

Despite the vast interest evidenced in the literature on amnestic MCI and its conversion to AD, the clinical significance of executive impairment in the early and differential diagnosis of MCI has been little explored [12,13]. EFs have been defined as a complex set of top-down cognitive processes that are necessary for the enactment of both self-regulation strategies and goal-directed behaviors [14,15,16]. These functions feature among the six core cognitive domains subject to neurocognitive decline, according to the Diagnostic Statistical Manual of Mental Disorders, 5th edition (DSM-5) [17], and seem to be associated with specific brain indices, such as prefrontal cortex (PFC) thickness, that could orient clinicians in predicting MCI prognosis [18]. Further changes in brain connectivity, especially the decreased integrity of the grey-matter network, have been observed in aged people in relation to memory, executive, and motor deficits [19]. A decline in the grey-matter network is associated with attentional/executive deterioration over the AD continuum, i.e., from preclinical disease to overt dementia [20].

### 1.2. The Relationship between Executive and Decision-Making Processes in the Neurocognitive Continuum

Of the several executive processes, decision-making (DM) skills emerge as some of the most commonly impaired in major neurocognitive disorders, which may lead to social, ethical, and health repercussions as patients progressively lose their judgement capacity and functional independence [13,21,22]. Similar difficulties are encountered in different clinical conditions (e.g., [23,24,25]) and, in general, involve cognitive and emotional abilities that underlie the choice between options in a given situation [26]. This multifactorial domain may be affected in late adulthood, AD [27], and frontotemporal dementia [28], being associated with poorer cognitive outcomes and emotion modulation issues. Besides aging and neurodegenerative diseases, specific conditions or life circumstances may lead to cognitive changes, emotional responses, and motivational shifts impacting the ways in which decision-relevant information is learned and manipulated [27,29,30]. Cognitive-based DM allows individuals to consciously monitor decisions and seems to prevail in conditions where individuals are able to estimate the risks and implications of their choices (as in most of routinized life activities), whereas emotion-based DM is supposed to occur especially under ambiguous contingencies as it entails emotional adjustments to uncertain surroundings [29]. Although current clinical tasks allow the assessment of DM within the executive domain [31], decisional skills can be also considered non-overlapping and relatively independent from mere executive control [32] as they also involve reward responsiveness [33] and affective processes [27]. Some authors recently endorsed a bipartite model of EFs by discriminating purely cognitive processing (cold EFs, involving lateral PFC) from emotionally driven processing (hot EFs, mostly related to ventromedial PFC and subcortical regions), which are engaged in specific DM tasks based on contextual information [34,35]. For instance, the underlying navigation behavior of DM is considered to be functionally cold, whereas DM under ambiguity is instead based on emotion-related experiences and reward encoding [34,35]. In addition to affect, attentional abilities and time perception may affect DM patterns, especially those concerning intertemporal choices that shape daily life [36]. DM can indeed refer to self-initiative in performing activities of daily living (e.g., physical exercise, financial management, living arrangements) and opting for medical treatments (e.g., feeding, taking prescribed medications, choosing between end-of-life care options). The ability to engage in such self-determination processes may indeed result in altering in both MCI and dementia, depending on the assessment tools and decision aids employed for both clinical and research purposes [37].

### 1.3. Interplay among Memory, Emotion-Based Learning, and Alterations in Complex DM

DM processes have also been proved to be interrelated with learning ones, as personally relevant decisions are mainly achieved through memory functions (e.g., encoding, storage, retrieval) and are continuously shaped by past experience [38]. Difficulties in the acquisition and consolidation of information represent markers of cognitive impairment that can also predict the conversion from MCI to dementia due to AD [39]. However, the link between episodic memory, active learning, and complex DM processes in patients affected by neurological conditions has been a matter of study and debate for the last three decades, also demonstrating the main role of emotions in the DM process [40,41,42].

Learning is a flexible, active memory sub-domain on which many intuitive, “gut-feeling”-like choices are based [42]. This process merges new and old information, and allows individuals to build up networks between and within different knowledge areas [43]. Several theories of value-based DM [44,45,46,47] state that determining which option to choose requires a calculation of the expected value or utility that will result from the consequences of such decision. This calculation facilitates a comparative process, allowing the agent to identify and pursue the option that leads to the highest expected value [48] and is also based on emotion regulation and intuition [30].

How such signals can be learned or acquired depends on the phasic activity of dopamine neurons (encoding a prediction error) on which the difference between expected and actual rewards depends [49]. Furthermore, abilities to relive past events on which to base one’s choices and behaviors relies on the emotional responses experienced during the encoding phase [50]. Emotions can also promote the acquisition and storage of central information (“what” happened in a situation) and instead dampen memory for some contextual information (e.g., “where”; “when”) [51], as in the case of aged-related hippocampal deficits.

Besides the study of emotion-based learning, some authors points to collecting both episodic memory and emotional recognition data to discriminate even mild AD-like impairment [52]. According to a broader intervention perspective, research on MCI focuses on improving emotional well-being, since mood disorders can co-occur with cognitive deficits [53], and the diagnosis of MCI is associated with the altered awareness of emotional states [54]. Moreover, cognitive reserve, referred to as a set of protective proxies against the onset or worsening of cognitive symptoms [55], represents the outcome of such experience-based learning processes that may be enhanced during later life, implying the maintenance of cortical and subcortical brain structures [56].

Overall, all these cognitive, emotional, and functional alterations observed in MCI demonstrate the need for caregiver support while taking relevant life decisions and thus require the adoption of a comprehensive approach to patients’ real-life issues and concerns [21,22].

### 1.4. Study Aims

Despite several efforts in identifying DM cognitive and emotional features and patterns over the aging continuum, the literature still lacks consensus about the criteria and tools that should be adopted to evaluate complex decisional processes in individuals suffering with MCI, along with neuropsychological factors that may explain the onset and progression of DM deficits throughout the course of time and neurocognitive deterioration [57].

In an attempt to bridge this gap, the present systematic review is aimed at investigating the relationship between DM and MCI with regard to the different models adopted for their operationalization and clinical assessment. By querying different databases and accounting for various DM classification systems, this work is meant to provide a more complex view of decision-makers affected by mild or early patterns of cognitive impairment, thus guiding professionals in implementing life-long learning, stimulation, and rehabilitation programs suited to the needs of patients and caregivers.

## 2. Materials and Methods

### 2.1. Search Strategies

This work was preregistered on the Open Science Framework (OSF) at https://osf.io/wvdrz, 22 June 2022. The associated project (accessible at https://osf.io/amvye/?view_only=, 13 March 2024), includes a section dedicated to Supplementary Materials. This section comprehensively details the methodology and data pertinent to our study.

Following the PRISMA (Preferred Reporting Items for Systematic Reviews and Meta-Analyses) guidelines [58,59], a systematic review was performed on all available research articles retrieved from six databases (i.e., Scopus; PubMed; Web of Science; Psychology and Behavioral Sciences Collection; Medline Complete; APA PsycInfo) covering the fields of psychology, medicine, and neuroscience. The query string was run on 18 May 2022 and included the search terms “MCI”, “mild cognitive impairment”, “decision making”, and “DM*”, combined with suitable Boolean operators (see Table 1). Given the differing settings of the database accessed, in Scopus and PubMed, the search was conducted by title and abstract jointly, whereas the remaining databases required independent searches for these two fields.

The query string output a total of 1689 documents that were collected in an ad hoc spreadsheet file. All duplicate articles were checked within each database and in cross-database comparison, and then deleted. Records with similar titles or abstracts were considered distinct.

Only full-text articles available in English were selected and subsequently screened for relevance, then cross-reviewed by two authors (i.e., FA and GG). The content reported in each title and abstract was thoroughly read to enable the exclusion of unrelated articles. No limitations were set by age, gender, or ethnicity.

### 2.2. Eligibility Criteria

**Figure 1:**
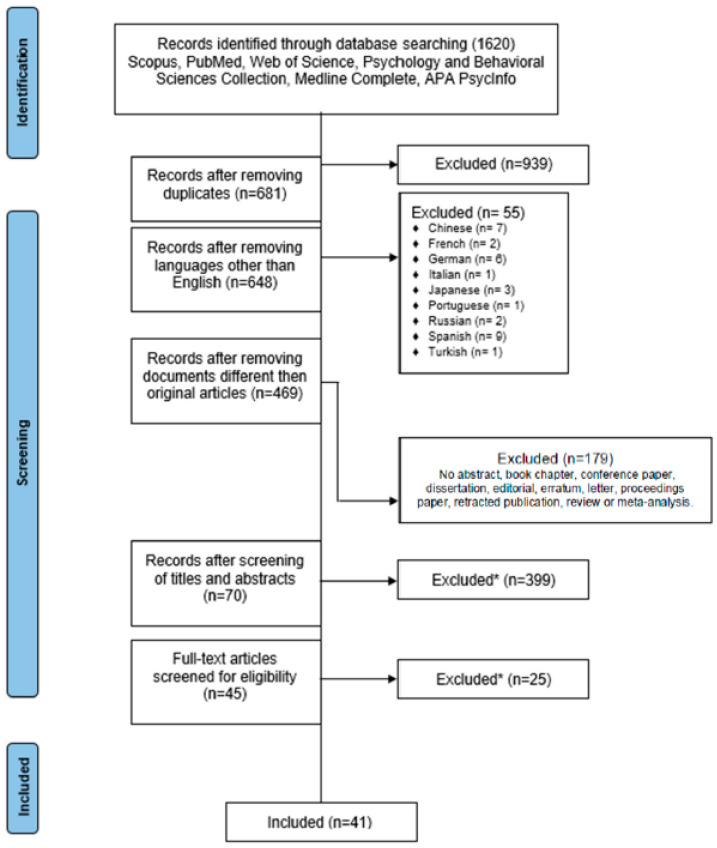
PRISMA flow diagram of the studies selections; * studies not complying with the eligibility criteria were excluded.

To be included in this review, the articles were required to meet six predefined eligibility criteria: (1) empirical studies involving MCI participants, regardless of their sex/gender and age; (2) studies where an assessment of DM was conducted; (3) original articles available in English; (4) full-text articles; (5) studies in which DM was discussed in relation to MCI or comparable patterns of cognitive impairment; and (6) studies primarily addressing patients’ DM and not clinicians’ or caregivers’ decisional processes. Accordingly, all the screened articles were scrutinized and independently reviewed by two authors (i.e., F.A. and G.G.), reaching an optimal inter-rater reliability (see Table 2). In cases of inconsistencies, these were solved by consensus or by consultation of an additional, independent rater (i.e., C.A.). All reasons for exclusion were noted (see Figure 1). The quality of the included studies was assessed by two of the authors (i.e., F.A. and C.A.) based on ten requirements provided by the JBI checklist, which are flexibly fit for different types of studies, notably observational (cross-sectional; retrospective case–control; prospective cohort) ones [60]. An appraisal score of at least 7/10 was considered acceptable for each article [61], depending on the clarity and soundness of information reported. As a result, all but two articles complied quality criteria, and their details were gathered and classified under the PICOS (Participants; Issues/Intervention; Comparisons; Outcomes; Study design) framework. This project and main data collection procedures were preregistered on Open Science Framework Registries (https://osf.io/wvdrz, accessed on 10 March 2024).

## 3. Results

All the studies included and assessed (see Table 3) were carried out during the period of 2005–2022, involving the investigation of one or more DM features in persons diagnosed with MCI or clinically equivalent stages. Most research (i.e., 30 out of 41 eligible studies) implemented a cross-sectional design, following a clinical–epidemiological approach. The total number of participants amounted to 5610 (including clinical subjects and healthy controls; women = 53.58%; age range = 60–82), in addition to 509 informants surveyed in two studies focused on patient–caregiver dyads [62,63].

MCI was not distinguished by etiology except for two studies that enrolled subjects affected by Parkinson’s disease–MCI [74] and MCI with Lewy Bodies [69]. Although 22 studies reportedly relied on Petersen’s criteria for its classification [2,3], only a few research works addressed amnestic MCI [83,88,90,91,93,94], while further subtypes were not considered for recruitment or diagnostic purposes in the remaining articles.

Different criteria used for the differential diagnosis between MCI and AD dementia mainly referred to the Alzheimer’s Disease and Related Disorders Association (NINCDS-ADRDA), the National Institute on Aging—Alzheimer’s Association (NIAA-AA), the fourth/fifth editions of the DSM, and cut-off scores from both the Mini-Mental State Examination (MMSE; [103]) and the Clinical Dementia Rating Scale (CDR; [104]).

The psychometric instruments included in the study protocols were mainly administered in a fixed order by well-trained healthcare professionals and research assistants operating in the clinical setting (i.e., hospitals; memory clinics; elderly care facilities). MCI evaluation was conducted using standardized screening protocols, domain-specific cognitive tests, questionnaires for the potential assessment of life autonomy and mood (depression; state and trait anxiety; apathy; further neuropsychiatric symptoms), and tasks targeted to the evaluation of DM abilities.

Only 8 out of 41 studies accounted for scores of global cognitive functioning and DM, whereas the remaining studies adopted a more extensive set of instruments. The most recurring screening tools consisted of the MMSE (29/41 studies) and the Montreal Cognitive Assessment (MoCA; 5/41 studies) [105], although additional batteries used in primary and secondary care settings have been reported. In view of the variety of tasks and questionnaires used to evaluate DM and its neuropsychological correlates, the subsections below illustrate the most recurring categories of DM investigated in cognitively impaired individuals.

### 3.1. Medical DM

The construction of medical DM involves multiple legal standards that require individuals to fully understand information in order to provide their consent (by means of hypothetical or figurative thinking), to weigh both costs and benefits from adhering to therapies, and to make reasonable choices pertaining to healthcare pathways. Accordingly, two observational studies detected in individuals with MCI a significant impairment of clinically relevant abilities, such as those relating to the comprehension and provision of informed consent to undergo medical treatment [93,94]. Deficits in memory and reasoning may affect medical DM differently as patients are required to recall the risks and benefits of each treatment option as well as to balance/compare them to justify the decision made [94]. While the aforementioned studies relied on the utilization of the Capacity to Consent Treatment Instrument (CCTI; [106]), other works adopted the Linguistic Instrument for Medical Decision-Making (LIMD) to measure the requirements for medical choices, finding altered responses in groups with different profiles of cognitive impairment [73,84,86]. Mental abilities covering clinical judgement appeared to be preserved in MCI despite lower DM scores for other areas of daily life, compared to those of cognitively spared individuals [88].

Patients suffering from neurodegenerative diseases presented different symptoms of cognitive decline and DM alterations, as AD typically threatens comprehension abilities and, conversely, PD results in difficulties in choosing suitable medical treatments [102].

Medical decisions could also be affected by further pathologies, such as progressive supranuclear palsy, involving slight cognitive dysfunctions and negative repercussions in the ability to provide consent for and make sound choices regarding research treatment [74].

### 3.2. Financial DM

Alterations in financial DM skills were observed in patients with MCI as progressive cognitive deterioration seems to reflect poorer abilities to manage finances. Indeed, when examined longitudinally, individuals with MCI exhibited a gradual loss in knowledge of assets/estate, investment decisions, basic monetary skills, and most notably, financial judgments [77]. Evidence of such a decline was obtained through the annual application of the Financial Capacity Instrument (FCI; [106]), in combination with cognitive screening tests, over a 6-year timespan.

Danesin et al. (2022) [64] showed that individuals suffering with MCI scored poorer than healthy individuals on both basic financial abilities (i.e., counting currencies; reading abilities; item purchase; bill payments; percentages) and high-level financial abilities (i.e., financial concepts; financial judgements). These skills were assessed using the Numerical Activities of Daily Living—Financial (NADL–F) short scale [107], allowing researchers to infer financial DM scores from the financial judgements subtest. In healthy controls, decisional competencies were found to be related to calculation and daily money management; however, subjects affected by neurodegenerative and neurological diseases presented a selective DM deficit not associated with impaired financial abilities. In addition to research focusing only on financial DM, we found that some authors examined this dimension as a part daily functioning (e.g., [63], and some others as related to medical DM. For instance, research based on risk aversion and temporal discounting paradigms highlighted the crucial role of subtle age-related decline in predicting the impairment of financial and medical DM [81], yielding results consistent with another study that detected deficits in both DM sub-domains in older individuals and patients with MCI [82]. However, it should be noted that a significant impairment in judgment skills may reflect advanced age even in the absence of MCI or AD diagnoses [87], prompting attention to DM impairment over preclinical stages of neurocognitive disorders.

### 3.3. Attentional, Visuo-Perceptual, and Linguistic DM Processes

Some studies examined the roles played by specific cognitive biases and neurocognitive indices that may occur in individuals with MCI, leading to dysfunctional patterns in judgement abilities.

Overall, cognitively impaired individuals seemed more prone to disadvantageous choices compared to aged-matched healthy controls [70]. To obtain differential profiles of patients based on vigilance, hypervigilance, and defensive avoidance, the authors evaluated DM performances in individuals with MCI and dementia and who were cognitively spared, while considering their cognitive and emotional statuses.

Another study assessed cognitive control in older adults with MCI compared to healthy ones using a perceptual decision-making task [75]. The authors focused on how the cognitive control index, being worse in the clinical sample, could be used to discriminate MCI profiles through a machine learning algorithm. Further scores obtained at a specific perceptual test indicated a greater tendency in patients with MCI to be distracted by irrelevant information, interfering with tasks set at a low cognitive load, and to deplete attentional responses in more challenging tasks, driven by bottom-up processes [66].

Through technology-based driving and crosswalk tasks (requiring visuospatial orientation, divided attention, executive, and DM skills), some authors observed the increased tendency for collisions and hesitation in cognitively impaired individuals in longer, two-lane, and trafficked roads [101]. Moreover, when undergoing eye-tracker-assisted tasks, patients with amnestic MCI showed fewer visuospatial judgment deficits when compared with others with mild AD [99]. Where perceptual and behavioral disorders were also present, as in MCI-LBD, impaired sensory processing was associated with slowness and executive issues [69].

Language-based DM was examined using appropriate experimental protocols as well as linguistic instruments that inquire into a patient’s comprehension abilities to meet the legal standards for adequate treatment choices.

One recent study investigated the decisions and attitudes concerning participation in clinical trials under different conditions of risks, highlighting differences only in the group with AD and non-MCI and healthy subjects [73], whereas previous research showed a progressive deterioration in these faculties becoming observable even in individuals with MCI [86]. Further evidence from factorial analyses revealed the critical role of verbal knowledge in predicting DM performances; in contrast, reading speed emerged as the most predictive measure deriving from the administration of a single test [84]. By means of neuroimaging techniques, an altered posterior cortical metabolism associated with verbal and medical reasoning skills was observed [90].

Besides possible deficits in linguistic medical DM capacity, patients also experienced semantic difficulties. For instance, when presented with words in semantic tasks, MCI and AD subjects performed less accurately and slower than healthy controls, and the scores obtained by both clinical groups at the semantic distance task appeared to be predictive of daily functioning [97]. Under ambiguous conditions, individuals with MCI also exhibited semantic interference, likely due more to executive impairment than to semantic representation loss [95].

### 3.4. DM under Risk/Ambiguity Conditions, and Preference Influence on Life Activities

In a drift diffusion-based study involving participants with MCI and mild AD, cognitive impairment emerged as critical in the occurrence of subtle deficits in a self-administered animacy task [67]. Prior to test administration, caregivers reported patients’ lack of confidence in everyday decisions that were consistent with the scores obtained from clinical subjects and their potential implications in the pursuit of everyday activities. In a further observational study [63], caregivers also provided information on elderly patients’ DM abilities through the Test of Practical Judgment—Informant (TOP-J-Informant) [108]. The use of this novel tool allowed scholars to shed light on key daily judgment issues (covering safety, medical, financial, and social DM) in cognitively impaired people, suggesting an association between older age and low education with worse decisional outcomes. However, previous research demonstrated advantages in adopting the Everyday Decision-Making Capacity (ACED; [98]) scale to assess understanding, appreciation, and reasoning abilities even in cases where patients showed self-neglect or a lack of independence in instrumental life activities [98]. To delve into the MCI neurocognitive profile, all these instruments were used in addition to or alternatively to DM-specific tasks, the results of which may carry implications for daily choices.

Galvin et al. (2020) [62] used a specific set-switching task that provided a measure of DM and problem-solving abilities, in relation to further basic and high-order functions. Such executive dysfunction, particularly evident in intertemporal DM tasks, could be imputable to frontal circuit degeneration [72]. In high-demanding situations, patients with MCI were more likely to pursue less convenient choices [79,80], yet patients facing these issues could benefit from targeted training on EFs and number processing [78].

Overall, although no major differences emerged between MCI and AD decisional strategies based on social norms [91], these groups tended to score lower than controls [76], which was also related to their increased rates of apathy [85]. Temporal lobe integrity could further contribute to differences in discounting responses [68], as individuals with defective retrieval of episodic information show reduced propensity for far-sighted decisions [83]. However, the literature concerning memory and DM performance showed some conflicting results, with no significant differences between MCI and controls based on reward sizes and no consistent associations between memory and time preference [96]. Further, olfaction (involved in both emotional and non-emotional memories), was not found to be associated with value-based DM and general cognition [100].

One of the few but significant differences observed between MCI and AD depended on the type of conditions the participants were exposed to, since DM deficits in the MCI group became more manifest only in cases of greater complexity and unpredictability [71]. Executive alterations emerged under risk and ambiguity, revealing difficulties encountered by patients with MCI in integrating decision-relevant information and learning from feedback to elect time-profitable options [89]. To assess decisional processes under uncertain conditions, several studies relied on the Iowa Gambling Task (IGT; [109] reported in [71,76,83,85,89,100]), whereas risk preferences were measured through the Game of Dice Task (GDT; [110] reported in [71,78,80]).

## 4. Discussions

The present review examined 41 eligible studies that involved the analysis of DM processes in participants who received a diagnosis of MCI or exhibited slight/subtle deficits when screened on cognitive tests. Based on the literature collected, the evaluation of mood status and daily independence was primarily conducted for MCI sample/group selection, rather than for probing possible associations with cognitive performances. DM examination was mainly covered within a more extensive neuropsychological (i.e., cognitive, emotional, and functional) assessment, in accordance with international guidelines [111], and further empirical evidence [1,112,113].

Despite only few studies having employed the MMSE as a stand-alone assessment tool, the results obtained from its single administration may misidentify some cognitive prodromes of dementia [114], requiring healthcare professionals to be cautious in interpreting global cognitive scores obtained by individuals with MCI. On the one hand, the diversity of clinical frameworks and instruments reported in the literature poses challenges to the comparison of MCI profiles and the development of etiological assumptions (e.g., “MCI due to AD”) [115]. On the other hand, the heterogeneity of these approaches, most likely due to the wide time range of study implementations, might contribute to a more thorough understanding of MCI manifestations and features. For instance, the MCI sub-types inferred from extensive assessment were not limited to the amnestic form but also resulted in non-amnestic, multiple domain patterns of impairment, of which executive MCI appeared to be prominent. These findings are consistent with those reported in a recent review on the early predictors of MCI, which demonstrated the co-occurrence and relative overlap of amnestic–executive abilities [116]. The investigation of MCI phenotypes, especially the dysexecutive one, may involve key clinical implications, such as the onset and development of specific cognitive symptoms, that can affect patients’ prognoses [11].

Although the scarcity of longitudinal approaches could represent a limitation for the prediction of possible conversion/reversion trajectories of MCI over time, further groups (e.g., AD-dementia; normal cognition) and/or caregivers were considered in most observational studies reviewed, allowing MCI scores to be compared with measures obtained from other subsets of participants. In addition to socio-demographics, clinical–anamnestic and neuroimaging data were gathered in some works, given the diagnostic and prognostic significance of biomarkers since pre-dementia stages [7,117]. Specifically, two articles (i.e., [67,69]) reported the advantages derived from combining clinical data and test scores through computational techniques, such as the drift diffusion model and machine learning algorithms, leading to accurate MCI classifications based on patients’ performances in perceptual and executive tasks. These models may also be helpful in discriminating individuals with further cognitive alterations (e.g., recognition deficits), providing an incremental value to more traditional diagnostic procedures [118].

With respect to DM assessment, the application of different tools (i.e., self-/informant-reported instruments vs. neuropsychological tests; laboratory-/paper-based vs. virtual tasks) highlighted the complexity of processes underlying the achievement of medical, financial, and other strategic life decisions.

Despite the prevailing focus on cognitive aspects of DM in the literature reviewed, real-life choices appear to be instead based on a combination of cognitive and emotional variables that concur with self-reflection and self-regulation in specific circumstances [119]. For instance, the increased apathy levels observed in cognitively impaired individuals [85] may be an additional factor of their worse decisional outcomes, as emotional processes and background experiences continuously shape motivation, cognition, and goal-oriented behaviors throughout an individual’s life [120]. These observations become even more relevant in research conducted on individuals with MCI, being commonly considered as aware (or even overly concerned) about their cognitive-functional status, in spite of some occasional judgement failures [121]. However, an appreciable decline in insight may be detected even in such patients [122], and this could also depend on the different types and number of cognitive domains affected.

Following a purely neurocognitive approach, MCI decisional performance has emerged as being associated or driven by specific sub-domains, i.e., (1) the learning and retrieval of verbal and visuospatial information; (2) the EFs of monitoring, feedback responding, and hypothetical/counterfactual thinking; (3) attentional-perceptual abilities, including information processing speed and shifting; and (4) linguistic skills, specifically comprehension and semantic accuracy. One or more of these functions being impaired results in MCI and dementia, often entailing poor decisional performance that can be explained according to the different domains affected.

Specifically, memory deficits might disrupt the recollection of information that is relevant to make personal choices, while executive dysfunctions could impact the cost-effectiveness and foresight of such decisions. Neuroimaging studies confirm the involvement of the hippocampus and orbitofrontal cortex in memory-guided decisions [123], along with PFC activation in executive ones [124]; however, further research is needed to delve into the involved in both memory- and executive-related DM performance.

The findings of the present review also pointed to overlapping patterns and features of linguistic–amnestic processes involved in DM as well as attentional–executive ones. Indeed, verbal comprehension deficits may prove to be affecting patients’ explicit knowledge, while perceptual–attentional skills may be considered prerequisites to undertake purposeful behaviors. A voxel-based study [125] showed not only the effects of aging in modulating the associations between volume in the aforementioned brain regions and cognitive scores but also provided an explanation for possible discrepancies between memory and executive performances, based on the view that episodic memory is concept-based whereas EFs are percept-based [126].

Despite the several cognitive features of DM, only one study among those included reported the effectiveness of an EF training program in improving ratio processes and DM under risky conditions in participants with MCI [78]. Similar approaches could be integrated with prevention/intervention strategies, such as social learning ones, that may debias judgements and decisions by observing the consequences of others’ choices and behaviors [127]. As social cognition may be affected by neurocognitive disorders [91], integrated, proactive programs should be tailored to the specific needs and concerns of patients with MCI, envisaging a baseline assessment of their social skills.

In real-life circumstances, DM is not limited to one’s capacity to rationally ascertain the odds of a risky event occurring but encompasses complex abilities aimed to adequately process and respond to high-demanding, uncertain situations. When assessed using tasks that can be run on both risky and ambiguous modes (e.g., IGT), patients with MCI yielded scores between that of controls and dementia patients. The most compelling evidence concerned specific difficulties experienced by MCI under ambiguity conditions, in addition to weak foresight that might translate into more impulsive life choices. According to the socioemotional selectivity theory [128], advancing age can indeed result in changes around processing experiences and setting priorities due to a greater propensity in later life to perceive time as narrower and thus to assign more value to emotionally charged, present-related choices [129].

It follows that the design and implementation of life-long learning programs should reflect progressive shifts in time preferences to effectively target motivation in elderly people, whether they be cognitively normal or are at risk of dementia.

Further explanations with respect to both memory and DM deficits in MCI and related treatment options can be provided by computational reinforcement learning (RL) algorithms to support learning [130], as computer science allows the deeper understanding of certain cognitive skills, such as risk prediction, learning, and calculation [131]. The study of RL has been used to infer brain functioning in numerous cortical and subcortical structures and to explain a vast range of related behaviors [132,133,134,135]. However, like many computational procedures, because of the lack of dimensionality of the approach, RL is reduced in effectiveness as the size of the problem increases [38,136].

The environment presents complex challenges, and the use of the new technologies can be an important contribution not only because they make it possible to evaluate DM processes by creating a functional environment where the user could potentially encounter real-world scenarios but researchers have also shown that it is a valid tool to evaluate activities of daily living [137]. The use of serious games created with augmented reality (AR) offers the possibility of reconstructing a user-friendly setting, allowing the simulation of everyday life situations, helping the elderly in the home environment while collecting their feedback [65]. Regarding potential effective treatments extended to the family system, the application of an intelligent assistive (AI) paradigm can reduce caregiver burden and compensate for the lack of human capital by empowering both caregivers and the overall care environment. This also translates into lesser repercussions on public health finances as technology-based interventions might offer greater chances for postponing or even limiting institutional care [138].

## 5. Concluding Remarks

In this review, we collected all available articles that, consistent with the inclusion criteria, delved into the relationship between DM and MCI, reporting the use of different appropriate tools for both screening and assessment purposes. The information retrieved also led to clinical considerations pertaining to training opportunities that may be seized to assist both elderly and patients with MCI with associated DM patterns of deficits (i.e., financial DM; medical DM; cognitive-based DM; DM under risky and/or uncertain conditions) before they result in a significant impairment of daily functioning.

Overall, despite the relative consistency of the results presented in the present review, the varying sample size of each study (ranging from a minimum of 23 to a maximum of 730 participants), the lack of etiological data, and a consensus to operationalize both MCI and DM constructs may constitute major limitations for the generalization of the findings obtained. Additionally, the diversity of methodological arrangements adopted in the literature for both diagnosis and treatment purposes could make it more difficult to achieve a quantitative approach from such results.

Nevertheless, the systematic collection and classification of studies on the manifold DM features and processes found in patients with MCI can provide clinicians and scholars with preliminary cues on cognitive–emotional correlates and predictors of DM impairment on which to build assessment and rehabilitation protocols tailored to patients’ and caregivers’ needs and feedback, as well as laying the foundation for future empirical studies on such emerging issues.

## Figures and Tables

**Table 1:** Databases and words used for the title and abstract searches.

Database	Following Words Researched within Titles and Abstracts
Scopus	(“MCI” OR “mild cognitive impairment”) AND (“decision making” OR “DM*”)
PubMed	
Web of Science	
Psychology and Behavioral Sciences Collection	
Medline Complete	
APA PsycInfo	

**Table 2:** Inter-rater agreement between two authors.

Cohen’s Kappa and Percentages of Agreement	
Judges	2
Total screened articles	55
Included articles	41
Both judges agree to include	41
Both judges agree to exclude	14
Only the first judge wants to include	0
Only the second judge wants to include	3
% of agreement	94.83%
Cohen’s kappa	0.87

**Table 3:** Summary table of articles included and discussed in our review.

Extraction Number	Authors	Publication Year	Reported Diagnoses of Participants	Comparison Groups	Types and Sub-Types of DM
1	Danesin et al. [64].	2022	MCI; PD; stroke	Yes	Financial DM
2	Ghorbani et al. [65].	2022	MCI	Yes	Problem-solving applying to everyday situations
3	Rabin et al. [63].	2022	Dementia; bvFTD; MCI; social communication disorder; VaD	No	Safety, medical, financial, social DM
4	He et al. [66].	2021	MCI	Yes	Visuo-perceptual DM
5	Karimi et al. [67].	2021	MCI/mild AD	Yes	Problem-solving applied to everyday situations
6	Galvin et al. [62].	2020	FTD; LBD; MCI; mild dementia; moderate dementia; severe dementia; VCID	Yes	Attention and problem-solving paradigms
7	Lempert et al. [68].	2020	MCI	Yes	Intertemporal DM
8	Revie et al. [69].	2020	MCI with Lewy body	Yes	Visuo-perceptual DM
9	Biella et al. [70].	2020	dementia; MCI	Yes	Cognitive-based DM
10	Sun et al. [71].	2020	AD; MCI	Yes	DM under risk/uncertainty
11	Geng et al. [72].	2020	dementia; MCI	Yes	Intertemporal DM
12	Stormoen et al. [73].	2019	AD; MCI	Yes	Medical and linguistic DM
13	Gerstenecker et al. [74].	2019	AD; MCI; PD; PDD; PD-MCI; PSP	Yes	Medical DM
14	He et al. [75].	2019	MCI	Yes	Perceptual DM
15	Jacus et al. [76].	2018	AD; MCI	Yes	DM under risk/uncertainty
16	Martin et al. [77].	2019	MCI; mild AD	Yes	Financial DM
17	Burgio et al. [78].	2018	MCI	Yes	DM under risky conditions
18	Pertl et al. [79].	2017	MCI	Yes	DM in everyday situations
19	Pertl et al. [80].	2015	MCI	Yes	DM under risky conditions
20	James et al. [81].	2015	n.r.	No	Financial and medical DM
21	Han et al. [82].	2015	MCI	Yes	Financial and medical DM
22	Bayard et al. [83].	2015	AD; MCI	Yes	DM under uncertainty
23	Stormoen et al. [84].	2014	AD; MCI	Yes	Medical and linguistic DM
24	Bayard et al. [85].	2014	AD; MCI	Yes	DM under uncertainty
25	Tallberg et al. [86].	2013	AD; MCI	Yes	Medical and linguistic DM
26	Boyle et al. [87].	2012	MCI	No	Financial and medical DM
27	Lui et al. [88].	2012	MCI; mild AD	Yes	Medical DM
28	Zamarian et al. [89].	2011	MCI	Yes	DM under risk/uncertainty
29	Griffith et al. [90].	2010	MCI	Yes	Medical DM
30	Bosch-Domènech et al. [91].	2010	MCI; mild AD	Yes	DM under uncertainty
31	Okonkwo et al. [92].	2008a	AD; MCI	Yes	Medical DM
32	Okonkwo et al. [93].	2008b	MCI	Yes	Medical DM
33	Okonkwo et al. [94].	2007	AD; MCI	Yes	Medical DM
34	Azuma et al. [95].	2013	MCI	Yes	Linguistic DM
35	Coelho et al. [96].	2017	MCI	Yes	Intertemporal DM
36	Kirchberg et al. [97].	2012	AD; aMCI	Yes	Linguistic DM
37	Lai et al. [98].	2008	MCI	Yes	DM in everyday situations
38	Laurens et al. [99].	2019	AD; aMCI	Yes	Visuo-perceptual DM
39	Ward et al. [100].	2017	AD; MCI	Yes	DM under risk/uncertainty
40	Dommes et al. [101].	2015	Mild/preclinical AD	Yes	Visuo-perceptual DM
41	Griffith et al. [102].	2005	AD; PD (incl. mild forms)	Yes	Medical DM

Acronyms and abbreviations: AD = Alzheimer’s disease; aMCI = amnestic mild cognitive impairment; DM = decision-making; bvFTD = frontotemporal degeneration—behavioral variant; FTD = frontotemporal degeneration; LBD = dementia with Lewy body; MCI = mild cognitive impairment; n.r. = not reported; PD = Parkinson’s disease; PDD = Parkinson’s disease dementia; PD-MCI = mild cognitive impairment due to Parkinson’s disease; PSP = progressive supranuclear palsy; VaD = vascular dementia; VCID = cerebral small vessel disease.

## Data Availability

Not applicable.

## Supplementary Materials

Tables with details of articles selected according to the implemented eligibility criteria according to PICOS guidelines, the checklist of the quality control and tables with details regarding the PRISMA selection are available on Open Science Framework Registries (https://osf.io/wvdrz, accessed on 22 June 2022).
